# Electrolyte design and interface engineering for high-voltage solid-state lithium batteries

**DOI:** 10.3389/fchem.2026.1840199

**Published:** 2026-05-13

**Authors:** Xianzheng Liu, Nashrah Hani Jamadon, Yueyue Yu, Liancheng Zheng, Rongji Tang

**Affiliations:** 1 College of Mechanical Engineering, Shandong Huayu University of Technology, Dezhou, Shandong, China; 2 Department of Mechanical and Manufacturing Engineering, Faculty of Engineering and Built Environment, Universiti Kebangsaan Malaysia, Bangi, Selangor, Malaysia

**Keywords:** electrolyte design, high voltage, interface engineering, lithium batteries, solid-state electrolyte

## Abstract

Solid-state lithium batteries (SSLBs) have attracted extensive attention as next-generation energy-storage systems because they offer improved safety and the possibility of coupling lithium metal anodes with high-energy cathodes. Among the many development directions of SSLBs, high-voltage systems are particularly important because they provide a direct pathway toward higher energy density. However, under high-voltage operation, typically above approximately 4.3 V versus Li^+^/Li but strongly dependent on cathode chemistry and state of charge, both the solid electrolyte and the electrode/electrolyte interface are subjected to severe electrochemical and structural challenges. Electrolyte oxidation, cathode-induced interfacial decomposition, space-charge effects, mechanical contact loss, and manufacturing difficulties jointly limit the practical performance of high-voltage SSLBs. This review systematically summarizes recent advances in electrolyte design for high-voltage SSLBs, covering inorganic solid electrolytes, polymer electrolytes, organic–inorganic composite electrolytes, gel polymer electrolytes, and quasi-solid-state electrolytes. In addition, the critical role of interface engineering is discussed with emphasis on cathode-side stabilization strategies, interphase regulation, and coating design. Finally, the major challenges and future research directions for high-voltage SSLBs are presented. The development of high-voltage SSLBs requires synergistic optimization of electrolyte chemistry, interfacial stability, and scalable processing strategies.

## 1 Introduction

The rapid growth of electric vehicles and large-scale energy-storage technologies has intensified the demand for batteries with both higher energy density and improved safety. Conventional lithium-ion batteries based on flammable liquid electrolytes have achieved enormous commercial success, but their safety risks and limited energy density have become increasingly difficult to ignore in advanced applications (Degen et al., 2023; Kalnaus et al., 2023; Wang et al., 2016). In this context, solid-state lithium batteries (SSLBs) are widely regarded as one of the most promising next-generation energy-storage technologies because solid electrolytes are generally nonflammable, thermally stable, and less volatile than liquid electrolytes, while also offering the possibility of suppressing lithium dendrite growth and enabling lithium metal anodes (Li et al., 2021; Liu et al., 2022; Manthiram et al., 2017). By combining solid electrolytes with lithium metal and high-energy cathodes, SSLBs may break through the energy-density ceiling of current liquid-electrolyte systems (Nolan et al., 2019).

In recent years, a major trend in SSLB research has been the shift from low-voltage cathodes toward high-voltage positive electrode materials. Although low-voltage cathodes such as LiFePO_4_ have been frequently used in early demonstrations, they do not fully exploit the intrinsic energy-density advantage of SSLBs. In contrast, high-voltage cathodes such as layered oxides, lithium-rich oxides, and LiNi_0.5_Mn_1.5_O_4_ can significantly raise the output voltage and practical energy density of solid-state batteries (Jang et al., 2022; Liu et al., 2026; Ohta et al., 2012; Yu et al., 2023). As a result, developing solid electrolytes that remain stable under high-voltage cathode environments has become one of the core tasks in SSLB research. It should be noted that “high voltage” in SSLBs should not be defined only by a fixed numerical threshold such as 4.3 V versus Li^+^/Li. This value is useful as a general reference, but the actual oxidative environment depends strongly on cathode chemistry, state of charge, transition-metal redox activity, surface oxygen reactivity, and the local structure of the composite cathode. For example, Ni-rich layered oxides, lithium-rich oxides, and spinel LiNi_0.5_Mn_1.5_O_4_ may impose different oxidative and catalytic conditions on the same electrolyte, even when their nominal cutoff voltages are similar. Therefore, electrolyte stability in high-voltage SSLBs should be evaluated in relation to the specific cathode/electrolyte interfacial environment rather than by voltage value alone.

However, high-voltage SSLBs face more complex problems than simply broadening the electrochemical stability window of the electrolyte. First, many conventional polymer electrolytes, especially PEO-based systems, are prone to oxidative decomposition under high-voltage conditions, leading to resistive interphase formation and rapid capacity fading (Yang et al., 2020). Second, highly delithiated cathode surfaces are usually much more reactive than their lithiated states and can accelerate electrolyte decomposition at the solid-solid interface (Chen et al., 2023; Janek and Zeier, 2023). Chen et al. emphasized that the practical application of all-solid-state lithium batteries with high-voltage cathodes is limited not only by the bulk stability of solid electrolytes, but also by their poor compatibility with cathode surfaces under realistic working conditions (Chen et al., 2023). Janek and Zeier further argued that the development of solid-state batteries is constrained by coupled chemical, interfacial, and engineering bottlenecks rather than by isolated material parameters (Janek and Zeier, 2023). Third, repeated phase transitions and volume changes in cathodes may induce interfacial contact loss, stress accumulation, and local current inhomogeneity during cycling (Lou et al., 2021). Finally, large-scale production of thin, continuous, and high-loading solid-state electrode/electrolyte structures remains difficult, especially for highly reactive or moisture-sensitive electrolyte systems (Schnell et al., 2018).

Therefore, the development of high-voltage SSLBs requires a systematic approach built on two tightly connected pillars: electrolyte design and interface engineering. The following sections first discuss the design principles and recent progress of representative electrolyte systems for high-voltage SSLBs, and then summarize interface-engineering strategies that are critical for stable operation.

**FIGURE 1:**
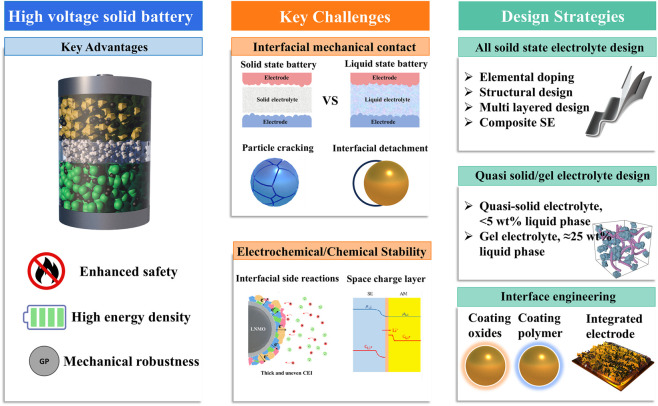
Schematic illustration of the advantages, key challenges, and electrolyte/interface design strategies for high-voltage solid-state lithium batteries.

Figure 1 summarizes the major advantages of high-voltage solid-state lithium batteries, the key obstacles limiting their practical application, and the corresponding design strategies discussed in this review.

## 2 Electrolyte design for high-voltage solid-state lithium batteries

In this review, high-voltage compatibility is discussed as a combined property of electrolyte chemistry, cathode reactivity, apparent electrochemical window, and practical interfacial stability in working cells. For high-voltage solid-state lithium batteries, different electrolyte systems should not be evaluated only by their nominal electrochemical stability windows or room-temperature ionic conductivities. A more useful comparison requires a unified framework that considers four coupled criteria: intrinsic oxidation tolerance at the cathode side, Li^+^ transport capability, interfacial contact and mechanical adaptability, and processing scalability. From this perspective, oxide electrolytes generally provide favorable oxidation resistance and mechanical rigidity, but their poor deformability and high-temperature processing often lead to large interfacial resistance. Sulfide electrolytes show outstanding ionic conductivity and good cold-pressing deformability, yet their limited oxidative stability and interfacial reactivity with high-voltage cathodes remain major concerns. Halide electrolytes offer a more balanced combination of cathode compatibility and Li^+^ transport, but their long-term stability, moisture sensitivity, and composition–property relationships still require further clarification. Polymer-based electrolytes are advantageous in flexibility and processability, although their oxidative stability and room-temperature conductivity are usually insufficient for demanding high-voltage cells. Organic–inorganic composite, gel, and quasi-solid-state electrolytes attempt to bridge these gaps by combining transport pathways, mechanical compliance, and interfacial adaptability, but they also introduce additional complexity in phase distribution, interphase evolution, and manufacturability. Therefore, the following discussion compares representative electrolyte families according to their high-voltage stability, ion-transport behavior, interfacial compatibility, and practical processing feasibility, rather than treating them as isolated material classes.

### 2.1 Inorganic solid electrolytes

Inorganic solid electrolytes are attractive for high-voltage SSLBs because they generally possess wider electrochemical stability windows and higher mechanical strength than polymer-based systems. The major inorganic electrolyte families include oxides, sulfides, and halides, each with distinct advantages and limitations.

#### 2.1.1 Oxide electrolytes

Oxide solid electrolytes are often considered promising for high-voltage applications because of their relatively strong oxidation resistance. Representative oxide electrolytes include garnet-type, NASICON-type, perovskite-type, and LISICON-type materials (Jian et al., 2017; Thompson et al., 2014). Ohta et al. demonstrated the electrochemical feasibility of all-solid-state lithium batteries using garnet-type oxide electrolytes, highlighting the potential of oxide systems in solid-state battery technology (Ohta et al., 2013). Nevertheless, oxide electrolytes also suffer from several limitations, including high sintering temperatures, rigid solid-solid interfaces, poor room-temperature contact with electrodes, and relatively high grain-boundary resistance.

To overcome these limitations, researchers have proposed multiple compositional and processing strategies. Qin et al. introduced nano-La_2_O_3_ into LLZTO and found that this additive promoted a more homogeneous electrolyte structure and created fast Li^+^ transport pathways through a three-dimensional continuous network (Qin et al., 2021). This work showed that grain-boundary engineering can simultaneously reduce interfacial resistance and improve microstructural uniformity. Feng et al. reported a low-temperature soldered all-ceramic lithium battery, demonstrating that appropriate interfacial modification can lower the processing temperature and help preserve electrode/electrolyte compatibility (Feng et al., 2021).

Atmosphere and thermal treatment also strongly affect oxide electrolyte quality. Huang et al. investigated the calcination growth of LLZO-based electrolytes at the atomic scale and clarified the evolution path of the Li^+^ conducting phase (Huang et al., 2020). Kim et al. showed that avoiding CO_2_ during annealing could improve the thermal stability of the LLZO/layered oxide interface, indicating that environmental control during processing is crucial for suppressing interfacial degradation (Kim et al., 2022). Huang et al. further demonstrated that manipulating the Li_2_O atmosphere during sintering accelerated densification and improved the ionic conductivity of dense LLZO-based electrolytes (Huang et al., 2019). More recently, Wang et al. combined theoretical guidance with ultrafast sintering to efficiently synthesize new garnet-type electrolytes, suggesting a route for accelerating oxide electrolyte discovery and optimization (Wang R. et al., 2020).

Overall, oxide electrolytes remain highly promising for high-voltage SSLBs because of their relatively high oxidation tolerance. However, their rigid nature, demanding sintering requirements, and persistent interfacial resistance still hinder broader application. Future advances will likely rely on combining composition optimization, atmosphere regulation, and low-temperature or rapid processing strategies.

#### 2.1.2 Sulfide electrolytes

Sulfide electrolytes have received extensive attention because of their high ionic conductivity and excellent deformability. Compared with oxides, sulfide electrolytes are easier to densify under cold pressing, making it easier to achieve intimate particle-particle contact within composite cathodes (Kim K. J. et al., 2021). These features make sulfides especially attractive for all-solid-state battery architectures requiring low interfacial resistance and high power density.

Despite these advantages, sulfide electrolytes are fundamentally challenged by limited oxidative stability under high-voltage conditions. Zhu et al. investigated the interfacial redox behavior of sulfide electrolytes and found that, under broader electrochemical windows, irreversible decomposition products such as Li_2_S and sulfur species can form at the interface, block ion transport, and increase impedance (Zhu et al., 2020). This means that the main challenge of sulfide electrolytes in high-voltage SSLBs is not only the thermodynamic stability of the bulk electrolyte, but also the dynamic evolution of the cathode/electrolyte interface during cycling.

To address these limitations, several structural and chemical modification approaches have been proposed. Gil-González et al. showed that chlorine substitution could synergistically improve the electrochemical behavior of sulfide electrolytes, demonstrating the importance of anion regulation in tuning interfacial stability (Gil-González et al., 2022). Wu et al. developed a core-shell structural design for sulfide electrolytes and demonstrated that this architecture improved both mechanical integrity and electrochemical performance (Wu et al., 2018). These results suggest that future sulfide electrolyte development must focus not only on maximizing conductivity but also on suppressing high-voltage interfacial decomposition through careful control of composition, defect chemistry, and local structure.

In summary, sulfide electrolytes remain among the most conductive solid electrolytes available, but their practical application in high-voltage SSLBs depends on effective interfacial stabilization and the mitigation of oxidative degradation.

#### 2.1.3 Halide electrolytes

Halide electrolytes have emerged as one of the most promising classes of solid electrolytes for high-voltage SSLBs. Compared with sulfides, halides generally show better oxidation resistance and improved compatibility with high-voltage cathodes, while also maintaining relatively fast Li^+^ transport (Wang Z.-Y. et al., 2023). These combined advantages have made halide electrolytes a rapidly expanding research frontier.

A landmark breakthrough was reported by Asano et al., who developed Li_3_YCl_6_ and Li_3_YBr_6_ as highly conductive halide solid electrolytes suitable for 4 V-class bulk-type all-solid-state batteries (Asano et al., 2018). This work opened a new avenue for high-voltage electrolyte design. Subsequently, aliovalent substitution became an important strategy for enhancing ionic conductivity. Park et al. introduced Zr^4+^ into mixed-metal halides and showed that the resulting solid electrolytes exhibited high ionic conductivity and high cutoff voltage because of vacancy generation and the creation of favorable three-dimensional Li^+^ transport pathways (Park et al., 2020).

However, conductivity enhancement alone is not sufficient. Kim et al. pointed out that the design of Li_3_MX_6_-type halide electrolytes must balance ionic transport, structural stability, and electrochemical robustness, because aliovalent substitution may also affect reduction stability and the overall materials chemistry (Kim K. et al., 2021). To address this challenge, Zhou et al. developed chloride-based halide electrolytes that enabled long-cycle-life 4 V ceramic all-solid-state batteries with high areal capacity (Zhou et al., 2022). More recently, Song et al. demonstrated that local lattice distortion in halide solid electrolytes can further improve high-voltage stability, underscoring the importance of fine structural tuning in addition to compositional design (Song et al., 2024).

Taken together, halide electrolytes are particularly attractive for high-voltage SSLBs because they combine good oxidation tolerance with favorable transport properties. Their future development will likely center on rational defect engineering, broader voltage compatibility, and long-term interfacial stability.

### 2.2 Polymer electrolytes

Polymer electrolytes are widely studied because of their flexibility, ease of processing, and better interfacial adaptability compared with rigid inorganic electrolytes (Wang X. et al., 2020). These features allow polymer-based systems to establish more intimate contact with electrode surfaces, which is beneficial for reducing interfacial resistance. However, their relatively low room-temperature ionic conductivity and limited oxidative stability remain major obstacles in high-voltage applications.

Beyond conventional PEO-based systems, a broader range of polymer hosts has also been explored for high-voltage solid-state lithium batteries. Fluorinated polymers such as PVDF-based are attractive because their fluorine-rich backbones generally provide better oxidative tolerance and improved compatibility with high-voltage cathodes (Li et al., 2025). Nitrile-containing polymers represented by PAN have also received attention due to their relatively wide electrochemical window and strong coordination with lithium salts, although their compatibility with lithium metal still requires careful interfacial regulation (Liu et al., 2024). In addition, methacrylate-based polymers such as PMMA are increasingly used in blended, crosslinked, or *in situ* polymerized electrolytes because they can help construct more amorphous and interfacially stable polymer networks (Yao et al., 2025). These results indicate that high-voltage polymer electrolyte design should not be limited to PEO modification alone, but should also consider alternative polymer chemistries with fluorinated, nitrile, or methacrylate functional characteristics.

Among the polymer systems studied so far, PEO-based electrolytes remain the most representative and most extensively investigated. Yang et al. examined the limiting factors of the electrochemical stability window of PEO-based solid polymer electrolytes and concluded that the terminal–OH groups play a key role in restricting oxidative stability (Yang et al., 2020). This finding offered a molecular-level explanation for the poor high-voltage tolerance of conventional PEO systems and motivated later efforts in molecular design and functional modification.

Several strategies have been proposed to improve polymer electrolytes for high-voltage SSLBs. Wang et al. designed a multilayer PEO electrolyte based on differentiated lithium salts, enabling one layer to better tolerate high voltage while another remained compatible with lithium metal (Wang et al., 2019). This study is particularly important because it reflects the practical asymmetry of battery interfaces: the cathode side and anode side impose different chemical requirements on the same electrolyte. Nie et al. developed an *in situ* polymerized interphase engineering strategy for high-voltage all-solid-state lithium-metal batteries, showing that interfacial polymerization can improve both contact and electrochemical stability (Nie et al., 2024). Zhao et al. further demonstrated that anion modulation can enable highly conductive and stable polymer electrolytes, confirming that salt chemistry is a powerful tool for regulating both transport and oxidation tolerance (Zhao et al., 2024).

Additional studies have emphasized polymer structure and localized plasticization. Bai et al. reported an *in situ* polymerized 1,3-dioxolane solid-state electrolyte with space-confined plasticizers for robust high-voltage Li/LiCoO_2_ batteries (Bai et al., 2023). Zhao et al. also demonstrated rechargeable lithium metal batteries with an in-built solid-state polymer electrolyte and a high-voltage, high-loading Ni-rich layered cathode (Zhao et al., 2020). Together, these studies show that high-voltage polymer electrolytes can be greatly improved through molecular design, salt engineering, multilayer construction, and *in situ* polymerization.

Although polymer electrolytes still face limitations in room-temperature conductivity and oxidation resistance, recent progress in fluorinated, nitrile-containing, methacrylate-based, and *in situ* polymerized systems shows that polymer electrolyte development for high-voltage SSLBs is becoming increasingly diversified beyond conventional PEO-centered designs.

### 2.3 Organic-inorganic composite electrolytes

Because neither inorganic nor polymer electrolytes can independently satisfy all the requirements of high-voltage SSLBs, organic-inorganic composite electrolytes have become an important intermediate strategy. Composite electrolytes aim to integrate the mechanical and electrochemical advantages of inorganic fillers with the flexibility and processability of polymers (Lv et al., 2019).

Composite designs may incorporate either inert fillers or ion-conductive fillers, but in high-voltage systems their function extends beyond simply enhancing ionic conductivity. Properly selected inorganic phases can also improve oxidative stability, reduce the direct exposure of vulnerable polymer segments to highly oxidizing cathode environments, and regulate cathode-side interfacial reactions. Yu et al. introduced Li^+^ containing continuous silica nanofibers into a composite polymer electrolyte and achieved enhanced Li^+^ conduction through more continuous transport pathways (Yu et al., 2019). Luo et al. used oxygen-vacancy-rich TiO_2_ microrods to improve both ionic conductivity and interfacial stability, indicating that filler surface chemistry can strongly affect the electrochemical performance of composite systems (Luo et al., 2023). Zhang et al. created vertically aligned nanoscale ceramic-polymer interfaces and demonstrated that more ordered ion-transport geometry can facilitate Li^+^ migration (Zhang et al., 2018). The ceramic fillers with high chemical stability and mechanical rigidity can help suppress local interfacial deterioration, while tailored filler surfaces or ordered multiphase architectures may promote the formation of more stable and less resistive interphases under high-voltage operation. Therefore, the design of organic–inorganic composite electrolytes for high-voltage solid-state batteries should be understood not only as a transport-pathway optimization strategy, but also as an approach to simultaneously improve voltage tolerance, mechanical integrity, and cathode/electrolyte compatibility.

Beyond simple filler dispersion, more sophisticated structural designs have also been explored. Jiang et al. reported an ultrathin bilayer composite electrolyte membrane with ceramic-in-polymer and polymer-in-ceramic architectures for high-voltage lithium metal batteries (Jiang et al., 2021). This type of asymmetric design allows different sections of the electrolyte membrane to perform different functions and thereby improves compatibility with both electrodes.

These studies indicate that composite electrolytes are not merely physical blends but carefully engineered multiphase systems. This high-voltage-oriented design concept is particularly evident in ceramic-rich composite electrolytes. Recently, Liu et al. reported a ceramic-dominant LLZTO–PEO–EC composite electrolyte for high-voltage solid-state batteries (Liu et al., 2026). The electrolyte delivered high ionic conductivity, a widened electrochemical stability window, and improved compatibility with high-voltage cathodes, highlighting the potential of ceramic-rich composite electrolytes for high-voltage SSLBs. Their further development will depend on the control of filler morphology, interfacial contact, dispersion homogeneity, and the continuity of long-range Li^+^ transport pathways.

### 2.4 Gel polymer and quasi-solid-state electrolytes

Gel polymer electrolytes and quasi-solid-state electrolytes offer another practical route toward high-voltage solid-state batteries. Compared with fully solid systems, they generally retain a certain degree of liquid-like interfacial adaptability while still providing better safety than traditional liquid electrolytes. For this reason, they are often regarded as transitional yet highly valuable platforms for high-voltage battery engineering.

Chen et al. developed a supramolecular-network gel polymer electrolyte with high ionic conductivity, demonstrating that carefully designed polymer networks can simultaneously provide mechanical support and efficient ion transport (Chen et al., 2022). Zhu et al. further reported an *in situ* three-dimensional cross-linked gel polymer electrolyte for long-cycle high-voltage lithium metal batteries, showing that network crosslinking is highly effective in stabilizing the electrochemical environment (Zhu et al., 2023). Kim et al. also demonstrated that sulfate additives can improve the electrochemical stability of PEO-based polymer electrolytes, which is especially relevant under high-voltage conditions (Kim et al., 2024).

In the quasi-solid-state domain, Zhou et al. developed an all-fluorinated polymer electrolyte for high-voltage lithium metal batteries, highlighting the beneficial role of fluorination in enhancing oxidative tolerance (Zhou et al., 2023). Qiu et al. used molecular anchoring at the electrode/electrolyte interface to regulate 4.7 V quasi-solid-state lithium metal batteries, illustrating how interfacial molecular design can compensate for the limitations of softer electrolyte systems (Qiu et al., 2023).

Although gel and quasi-solid-state electrolytes do not always meet the strictest definition of all-solid-state batteries, they offer important insights into interface adaptation, voltage stabilization, and practical cell design for high-voltage applications.

Based on this comparative framework, Table 1 summarizes representative electrolyte systems for high-voltage solid-state lithium batteries in terms of their intrinsic voltage tolerance, Li + transport characteristics, interfacial adaptability, processing advantages, and remaining limitations. This comparison highlights that no electrolyte family is universally superior; instead, practical high-voltage SSLBs require electrolyte selection and modification according to the specific cathode chemistry, operating voltage, interfacial environment, and manufacturing route.

**TABLE 1:** Comparative summary of representative electrolyte systems for high-voltage solid-state lithium batteries.

Electrolyte system	Representative examples	Comparative assessment and design implication	Design focus/Recommended direction
Oxide solid electrolytes	Garnet-type LLZO/LLZTO; NASICON-type electrolytes	Good oxidation stability and mechanical strength, but rigid interfaces and high-temperature processing cause poor contact and high interfacial resistance	Improve interfacial contact through surface modification, low-temperature processing, or composite designs
Sulfide solid electrolytes	Argyrodite-type and related sulfide electrolytes	High ionic conductivity and good deformability, but poor oxidative stability at high voltage and possible interfacial decomposition	Use cathode coatings, buffer layers, or artificial interphases to suppress oxidation
Halide solid electrolytes	Li_3_YCl_6_, Li_3_YBr_6_, and substituted chloride/bromide electrolytes	Better compatibility with high-voltage cathodes than many sulfides, but long-term stability and scalable synthesis remain challenging	Optimize composition, defect chemistry, and cathode-side interfacial durability
Polymer electrolytes	PEO-, PVDF-, PAN-, PMMA-based and *in situ* polymerized systems	Flexible and easy to process, but limited room-temperature conductivity and insufficient intrinsic high-voltage stability	Improve oxidation tolerance through fluorination, salt design, crosslinking, and multilayer structures
Organic-inorganic composite electrolytes	Polymer electrolytes containing LLZTO, TiO_2_, silica nanofibers, or other ceramic fillers	Combine polymer flexibility with ceramic-assisted ion transport, but filler aggregation and discontinuous pathways may reduce performance	Control filler dispersion, ceramic–polymer interfaces, and continuous Li + pathways
Gel polymer and quasi-solid-state electrolytes	Cross-linked gel electrolytes; fluorinated quasi-solid electrolytes	Good wettability and interface adaptability, but residual liquid components may affect long-term safety and durability	Strengthen polymer networks and immobilize liquid components for stable high-voltage operation
Ceramic-rich composite electrolytes	LLZTO-dominant polymer–ceramic composite electrolytes	Offer better oxidation tolerance than polymer-rich systems while retaining some interfacial flexibility, but processing uniformity remains difficult	Balance ceramic continuity, polymer-assisted contact, and scalable membrane fabrication

The comparison in Table 1 also indicates that the reported electrolyte strategies should be evaluated through trade-offs rather than single performance metrics. Oxide electrolytes are attractive for high-voltage stability, but their rigid interfaces and high-temperature processing limit intimate contact with composite cathodes. Sulfide electrolytes provide high ionic conductivity and favorable deformability, but these benefits are offset by oxidative instability and the need for protective cathode coatings. Halide electrolytes offer a promising balance between cathode compatibility and ion transport, yet their long-term durability, moisture tolerance, and scalable synthesis remain insufficiently established. Polymer and composite electrolytes improve processability and interfacial adaptability, but they often face compromises among oxidative stability, mechanical strength, room-temperature conductivity, and long-term interphase control. Therefore, electrolyte design for high-voltage SSLBs should move from maximizing a single property toward balancing voltage tolerance, Li^+^ transport, interfacial compatibility, mechanical integrity, and manufacturing feasibility.

## 3 Interface engineering for high-voltage solid-state lithium batteries

Even when a solid electrolyte exhibits satisfactory bulk ionic conductivity and apparent electrochemical stability, the actual performance of a high-voltage SSLB is often dictated by the cathode/electrolyte interface. Under high-voltage operation, the delithiated cathode surface becomes highly reactive and can trigger a series of degradation processes, including electrolyte oxidation, impedance buildup, unstable interphase growth, gas evolution, and interfacial contact loss (Janek and Zeier, 2023). Therefore, interface engineering is not a secondary optimization step, but a central requirement for controlling charge transfer, Li^+^ redistribution, chemical decomposition, and mechanical contact evolution in high-voltage SSLBs.

The fundamental origin of cathode/electrolyte interfacial degradation is the mismatch among electronic structure, Li^+^ chemical potential, chemical reactivity, and mechanical deformation at the buried solid–solid interface. First, electronic structure mismatch between a highly delithiated high-voltage cathode and the solid electrolyte may promote parasitic charge transfer across the interface. When the electrolyte cannot electronically withstand the oxidizing potential of the cathode surface, local electron leakage or hole transfer can trigger oxidative decomposition and the formation of resistive interphase products. This process is especially severe when conductive carbon, surface defects, or electronically leaky interphases provide additional pathways for parasitic reactions.

Second, differences in Li chemical potential and defect chemistry between the cathode and the solid electrolyte can induce space-charge layers near the interface. Such local redistribution of Li ions and charged defects may lead to Li^+^ depletion or accumulation, local potential gradients, and increased interfacial resistance. Therefore, a solid electrolyte with high bulk ionic conductivity may still show poor cell performance if interfacial Li^+^ transport is blocked by space-charge-induced transport bottlenecks.

Third, high-voltage cycling introduces strong chemo-mechanical coupling at the cathode/electrolyte interface. Repeated delithiation and lithiation of the cathode can cause lattice strain, surface reconstruction, volume change, and local stress accumulation. These mechanical changes may weaken solid–solid contact, create microvoids or interfacial cracks, and expose fresh reactive surfaces to the electrolyte. Once chemical decomposition and mechanical contact loss occur simultaneously, the interface may enter a self-accelerating degradation process: interphase growth increases impedance, stress concentration worsens contact loss, and newly exposed surfaces further promote side reactions. Therefore, interfacial degradation in high-voltage SSLBs should be understood as a coupled electronic–ionic–chemical–mechanical failure process rather than as simple electrolyte oxidation alone.

From a mechanistic perspective, interface engineering in high-voltage SSLBs can be understood through four coupled functions: chemical/electronic blocking, Li^+^ transport preservation, chemo-mechanical accommodation, and controlled interphase formation. First, the interface must suppress direct electron transfer and oxidative decomposition between the highly delithiated cathode surface and the solid electrolyte. Second, it should maintain continuous Li^+^ transport across the cathode/electrolyte boundary without introducing excessive interfacial resistance. Third, it must tolerate local stress, volume change, and contact degradation during repeated high-voltage cycling. Finally, it should guide the formation of a stable and thin interphase rather than allow uncontrolled decomposition products to accumulate. Therefore, coatings, buffer layers, and *in situ* interphase regulation should not be viewed as independent empirical strategies, but as different ways to control charge transfer, chemical reactivity, mechanical contact, and interphase evolution at the buried cathode/electrolyte interface.

Within this framework, cathode surface coatings mainly serve the function of chemical and electronic blocking while preserving Li + transport. An ideal coating layer should be sufficiently ion-conductive to avoid large polarization, but electronically insulating enough to suppress electron leakage from the delithiated cathode surface to the electrolyte. In high-voltage cathodes, surface reconstruction, transition-metal redox activity, and oxygen-related reactivity can accelerate electrolyte oxidation. Therefore, coatings do not merely act as passive physical barriers; they regulate the local electrochemical environment by reducing direct cathode/electrolyte contact, limiting parasitic charge transfer, and stabilizing the interfacial reaction pathway. Wang et al. emphasized that interface coating design is crucial for dynamic voltage stability in solid-state batteries (Wang Y. et al., 2023). Liang et al. further reported that lithium niobium oxide coatings could stabilize the interface between Ni-rich cathodes and PEO-based electrolytes, thereby improving the cycling performance of high-voltage all-solid-state batteries (Liang et al., 2020).

Catholyte and buffer layers provide a second type of interface regulation by redistributing chemical reactivity away from the most vulnerable cathode/electrolyte contact. Unlike dense surface coatings, buffer layers can act as chemically adaptive regions that absorb interfacial mismatch, regulate decomposition pathways, or participate in the formation of a more stable cathode/electrolyte interphase. For example, Wang et al. introduced a sacrificial C60-containing catholyte buffer layer in high-voltage LATP-based all-solid-state batteries (Wang et al., 2024). The preferential oxidation of C60 promoted the formation of a thinner and more stable interphase, thereby protecting polymer-containing components from uncontrolled decomposition. This strategy shows that buffer layers can be designed not only to separate incompatible phases, but also to guide interphase chemistry through controlled sacrificial reactions.

In polymer-based and quasi-solid-state systems, *in situ* interphase formation and molecular anchoring mainly address the problems of intimate contact, local chemical compatibility, and stress accommodation. Because soft electrolyte components can better conform to cathode surfaces, their interfacial stability is strongly influenced by local polymer chemistry, salt decomposition, and molecular interactions. Nie et al. showed that *in situ* polymerized interphase engineering can improve high-voltage lithium-metal batteries by creating a more stable and intimate electrode/electrolyte interface (Nie et al., 2024). Qiu et al. further demonstrated that molecular anchoring can regulate the interface in quasi-solid-state 4.7 V batteries (Qiu et al., 2023). These approaches indicate that interfacial stability is not determined only by the bulk electrolyte composition, but also by the local bonding, wetting, and interphase-growth behavior at the cathode/electrolyte boundary.

Overall, interface engineering in high-voltage SSLBs should be understood as a mechanism-driven strategy for regulating charge transfer, chemical reactivity, Li^+^ transport, and mechanical contact at the cathode/electrolyte interface. Cathode coatings mainly suppress direct electronic and chemical reactions, buffer layers regulate interphase formation and reaction pathways, while *in situ* interphase and molecular regulation improve local contact and chemo-mechanical adaptability. Future progress will depend on integrating these strategies according to the specific cathode chemistry, electrolyte type, and operating voltage, rather than applying interfacial modifications as isolated empirical treatments.

## 4 Challenges, trade-offs, and future priorities

Despite substantial progress, several critical challenges still prevent the practical deployment of high-voltage solid-state lithium batteries. A central issue is that most reported strategies improve one aspect of cell performance while introducing new limitations elsewhere. No single electrolyte family currently satisfies all the requirements of high oxidative stability, fast Li^+^ transport, intimate interfacial contact, mechanical robustness, and scalable manufacturability. Oxide electrolytes are chemically and electrochemically robust at the cathode side, but their rigidity, high sintering temperature, and poor deformability make low-resistance solid–solid contact difficult. Sulfide electrolytes provide high ionic conductivity and favorable cold-pressing processability, but their limited oxidative stability requires additional coatings or buffer layers under high-voltage operation. Halide electrolytes are promising for high-voltage cathodes, yet their long-term interfacial durability, moisture sensitivity, and scalable synthesis remain insufficiently established. Polymer and composite electrolytes are attractive for flexible processing and interfacial adaptation, but they often face compromises among room-temperature conductivity, oxidative stability, mechanical strength, and long-term interphase control. Therefore, the practical value of each electrolyte strategy should be judged by its overall balance among stability, transport, interfacial compatibility, and manufacturability rather than by a single reported parameter.

The cathode/electrolyte interface remains the central bottleneck in high-voltage solid-state lithium batteries. As discussed above, interfacial degradation originates from coupled electronic, ionic, chemical, and mechanical mismatches rather than from electrolyte oxidation alone. Therefore, future studies should not evaluate interfacial stability only from bulk electrolyte properties, but should consider cathode surface reactivity, space-charge effects, interphase evolution, and contact degradation under repeated high-voltage cycling.

Another important challenge is the interpretation of the electrochemical stability window. In many studies, the oxidative stability of solid electrolytes is evaluated by linear sweep voltammetry, cyclic voltammetry, or related measurements using inert or blocking electrodes. However, the stability window obtained from these simplified measurements should be regarded as an apparent stability window rather than a direct indicator of practical high-voltage compatibility. The measured value can be strongly influenced by electrode configuration, scan rate, temperature, stack pressure, contact area, and cutoff-current criteria. More importantly, inert-electrode tests do not reproduce the chemically active environment of real composite cathodes, where highly delithiated cathode particles, conductive carbon, electrolyte particles, local electronic leakage, and buried solid–solid interfaces coexist. Therefore, high-voltage stability should be evaluated by combining apparent electrochemical window measurements with full-cell cycling, constant-voltage holding tests, impedance evolution, and post-cycling interfacial characterization.

Manufacturing and scalability issues must also be considered more seriously. Many laboratory-scale demonstrations still rely on complex synthesis methods, moisture-sensitive electrolytes, ultrathin coatings, carefully controlled stack pressure, or low-mass-loading electrodes. These conditions are useful for proving scientific concepts, but they may not directly translate into practical cell manufacturing. For example, cathode coatings must be uniform, ion-conductive, electronically insulating, and compatible with large-scale powder processing. Composite electrolytes require homogeneous filler dispersion, continuous Li^+^ pathways, controllable membrane thickness, and stable ceramic–polymer interfaces. Gel and quasi-solid-state systems require careful control of residual liquid components to avoid compromising safety and long-term durability. In addition, many high-voltage solid-state battery studies are still performed in small laboratory cells, whereas practical implementation requires validation under high cathode loading, limited excess lithium, controlled electrolyte thickness, realistic stack pressure, and pouch-cell-relevant fabrication conditions.

Looking ahead, future research should move from broad material screening toward problem-oriented electrolyte–interface co-design. In our view, the most critical bottleneck is not simply the lack of electrolytes with wide apparent electrochemical stability windows, but the difficulty of maintaining stable cathode/electrolyte interfaces under realistic high-voltage and long-cycle conditions. Four priorities are particularly important. First, high-voltage stability should be evaluated in a cathode-dependent manner, because Ni-rich layered oxides, lithium-rich oxides, and spinel LiNi_0.5_Mn_1.5_O_4_ impose different oxidative, catalytic, and mechanical environments on the electrolyte. Second, interface engineering should shift from empirical coating selection to mechanism-guided interfacial protection, including ion-conductive and electronically insulating coatings, gradient buffer layers, and controlled sacrificial interphases. Third, electrolyte development should be integrated with interface design; halide electrolytes, oxide-rich composite electrolytes, and chemically designed polymer/composite interphases appear particularly promising, provided that their long-term interfacial durability can be demonstrated. Fourth, practical validation should become a central criterion, with more attention paid to high-loading cathodes, realistic stack pressure, scalable membrane fabrication, and pouch-cell-level evaluation. Overall, the field should move toward cathode-specific, interface-centered, and manufacturing-compatible design principles.

## 5 Conclusion

High-voltage solid-state lithium batteries represent a key direction for next-generation high-energy and high-safety electrochemical storage systems. Their development depends not only on discovering solid electrolytes with wider electrochemical stability windows, but also on the coordinated optimization of ionic conductivity, interfacial compatibility, structural stability, and manufacturability. Oxide, sulfide, halide, polymer, composite, gel, and quasi-solid-state electrolytes each provide distinct opportunities for high-voltage operation, yet each also presents characteristic limitations. At the same time, interface engineering plays a decisive role in stabilizing the cathode/electrolyte interface and enabling long-cycle performance under high-voltage conditions. Among these factors, cathode-dependent interfacial stability under realistic high-voltage cycling should be regarded as the central bottleneck for practical implementation. Overall, the future of high-voltage SSLBs will rely on cathode-specific electrolyte selection, mechanism-guided interface engineering, and scalable cell manufacturing rather than on single-parameter electrolyte optimization alone.
